# Increasing number of components of the metabolic syndrome and cardiac structural and functional abnormalities – cross-sectional study of the general population

**DOI:** 10.1186/1471-2261-7-17

**Published:** 2007-06-07

**Authors:** Ana Azevedo, Paulo Bettencourt, Pedro B Almeida, Ana C Santos, Cassiano Abreu-Lima, Hans-Werner Hense, Henrique Barros

**Affiliations:** 1 Department of Hygiene and Epidemiology, University of Porto Medical School, Porto, Portugal; 2 Department of Internal Medicine, Hospital de S. João and University of Porto Medical School, Porto, Portugal; 3 Department of Cardiology, University of Porto Medical School, Porto, Portugal; 4 Institute of Epidemiology and Social Medicine, University of Muenster, Muenster, Germany

## Abstract

**Background:**

We aimed to assess whether we could identify a graded association between increasing number of components of the metabolic syndrome and cardiac structural and functional abnormalities independently of predicted risk of coronary heart disease by the Framingham risk score.

**Methods:**

We conducted a cross-sectional study on a random sample of the urban population of Porto aged 45 years or over. Six hundred and eighty-four participants were included. Data were collected by a structured clinical interview with a physician, ECG and a transthoracic M-mode and 2D echocardiogram. The metabolic syndrome was defined according to ATPIII-NCEP. The association between the number of features of the metabolic syndrome and the cardiac structural and functional abnormalities was assessed by 3 multivariate regression models: adjusting for age and gender, adjusting for the 10-year predicted risk of coronary heart disease by Framingham risk score and adjusting for age, gender and systolic blood pressure.

**Results:**

There was a positive association between the number of features of metabolic syndrome and parameters of cardiac structure and function, with a consistent and statistically significant trend for all cardiac variables considered when adjusting for age and gender. Parameters of left ventricular geometry patterns, left atrial diameter and diastolic dysfunction maintained this trend when taking into account the 10-year predicted risk of coronary heart disease by the Framingham score as an independent variable, while left ventricular systolic dysfunction did not. The prevalence of left ventricular diastolic dysfunction, and the mean left ventricular mass, left ventricular diameter and left atrial diameter increased significantly with the number of features of the metabolic syndrome when additionally adjusting for systolic blood pressure as a continuous variable.

**Conclusion:**

Increasing severity of metabolic syndrome was associated with increasingly compromised structure and function of the heart. This association was independent of Framingham risk score for indirect indices of diastolic dysfunction but not systolic dysfunction, and was not explained by blood pressure level.

## Background

The metabolic syndrome is associated with an increased risk of coronary heart disease [1] which in turn is one of the major causes of heart failure and left ventricular systolic and diastolic dysfunction. Furthermore, hypertension, obesity, and diabetes mellitus have been shown to be independently associated with an increased risk of heart failure, regardless of coronary heart disease [2-8]. Therefore, an association between the presence of the metabolic syndrome and the occurrence of different stages of heart failure seems plausible.

Given the increasing prevalence of heart failure and its consequences – impaired quality of life [9], frequent hospital admissions and high case fatality rate [10] – it appears of importance to assess how its occurrence is associated with the highly prevalent [11] metabolic syndrome. We conceptualise a potential causal pathway for this association in which increasing severity of the metabolic syndrome, quantified as increasing concurrence of its single components, leads to increasingly compromised structure and function of the heart.

There has been much debate about the putative usefulness of the metabolic syndrome in cardiovascular risk prediction, namely whether it adds information to that provided by its individual components and, on the other hand, whether it adds to alternative risk prediction tools [12-14], among which the Framingham risk score [15] is the most widely used.

The aim of this study was therefore to assess whether we could identify a graded association between increasing number of components of the metabolic syndrome and cardiac structural and functional abnormalities in a cross-sectional study of urban Portuguese adults. Additionally, we intended to investigate whether this association was independent of predicted risk of coronary heart disease by the Framingham risk score.

## Methods

As part of a health and nutrition survey of a representative sample of the adult population of Porto, Portugal, all participants aged ≥ 45 years and recruited from January 2001 to December 2003 were assessed with a systematic evaluation of parameters of cardiac structure and function. A detailed description of the study has been published [16]. In brief, random digit dialling was used to select households followed by simple random sampling to select one subjects aged ≥ 18 years within each household Refusals were not substituted. The proportion of participation was 70% [17]. The local ethics committee approved the study and participants provided written informed consent.

Participants were invited to visit our Department for an interview, which included questionnaires on demographic, behavioural and clinical data. After a 12-hour fast, a venous blood sample was collected. Serum glucose level was determined using routine enzymatic methods, and cholesterol and triglyceride levels were determined using standard enzymatic colorimetric methods [18,19]. HDL cholesterol levels were determined after precipitation of apolipoprotein B-containing lipoproteins [20]. Anthropometric evaluation included waist circumference measurement to the nearest centimeter, midway between the lower limit of the rib cage and the iliac crest, with the subject standing using a flexible and non-distensible tape [21]. A spirometry and a resting 12-lead ECG were performed. Ischemic heart disease was defined as self-reported medical diagnosis of angina pectoris or myocardial infarction or pathologic Q waves on at least two adjacent leads on resting 12-lead ECG. Chronic lung disease was defined as history of chronic bronchitis or a diagnosis of moderate-severe obstructive (FEV1 < 70% of predicted) or restrictive (vital capacity < 70% of predicted) syndrome on spirometry. A few days after the first interview, a structured clinical interview by a physician, a cardiovascular physical examination and a transthoracic echocardiogram with pulsed Doppler evaluation of transmitral inflow were performed. Hypertension was defined as blood pressure ≥ 140/90 mmHg or being under anti-hypertensive medication. Blood pressure was measured after a 10-minute rest, with no tight clothes. The mean of two measurements was registered. Diabetes mellitus was considered as self-reported or fasting venous blood glucose ≥ 126 mg/dl [22]. Participants who were under antihypertensive or antidiabetic medications were considered to have high blood pressure or high glucose levels, regardless of current blood pressure or serum glucose [23,24]. The predicted 10-year risk of coronary heart disease was calculated for participants aged less than 75 years who had no coronary heart disease, according to the Framingham Heart Study risk prediction score using risk factor categories [15]. Models based on LDL cholesterol were used.

Echocardiograms were done by four cardiologists, using the same equipment (HP Sonos 5500) and recorded on videotape for later review by a single experienced cardiologist (C.A.L.), blinded to clinical data. Measurements of wall thicknesses and chamber dimensions were obtained according to the Penn convention and left ventricular mass was calculated as 1.04 [(interventricular septum thickness + LV end-diastolic diameter + posterior wall thickness)^3 ^- (LV end-diastolic diameter)^3^] - 13.6 [25]. Left ventricular mass, wall thicknesses and chamber diameters were indexed to height. Left ventricular mass was also indexed to height^2.7 ^and fat-free mass, estimated according to the equations reported by Kuch et al [26] using height and weight, in order to assess whether alternative indexation methods influenced the results. Left ventricular (LV) systolic function was assessed by the subjective impression of the operating cardiologist later validated by one single experienced cardiologist. Left ventricular ejection fraction was estimated by Simpson's rule. Left ventricular systolic dysfunction (LVSD) was defined as either ejection fraction < 45% or by eyeballing, this being the only parameter when ejection fraction could not be calculated (44 participants). In participants in sinus rhythm, the peak E wave/peak A wave ratio (E/A), E wave deceleration time (DT) and isovolumetric relaxation time (IVRT) were used to define the presence of diastolic dysfunction according to the European Society of Cardiology [27]: IVRT > 100 ms and/or E/A < 1.0 and DT > 220 ms if aged < 50 years; IVRT > 105 ms and/or E/A < 0.5 and DT > 280 ms if aged = 50 years.

Participants were classified according to the stages of heart failure defined by the American College of Cardiology and American Heart Association [28]: Stage A – high risk of heart failure, including hypertension, diabetes mellitus, metabolic syndrome, coronary heart disease, smoking, excessive alcohol intake; Stage B – asymptomatic heart disease, including left ventricular systolic dysfunction, left ventricular dilatation, moderate-severe valvular heart disease and left ventricular hypertrophy; Stage C – symptomatic heart failure, including heart failure symptoms or signs plus any of the abnormalities described in stage B, with the exception of left ventricular hypertrophy which was considered to be responsible for those symptoms only in the absence of chronic lung disease. Dyspnoea, orthopnoea, nocturnal paroxysmal dyspnoea, evening lower limb oedema, jugular venous distension and rales were considered related to heart failure when at least two of them were present. Lower limb oedema was disregarded if there were signs of chronic venous insufficiency.

Features of the metabolic syndrome were defined according to the Adult Treatment Panel III of the National Cholesterol Education Program (ATPIII-NCEP) [23]:

1. Waist circumference > 102 cm in men and > 88 cm in women;

2. Fasting serum triglycerides ≥ 150 mg/dl;

3. High-density lipoprotein (HDL) cholesterol < 40 mg/dl in men and < 50 mg/dl in women;

4. High blood pressure: systolic blood pressure ≥ 130 mmHg and/or diastolic blood pressure ≥ 85 mmHg or antihypertensive drug treatment;

5. High glucose levels: fasting serum glucose ≥ 110 mg/dl or clinical diagnosis of diabetes.

### Statistical analysis

Due to missing data on key variables for the definition of the metabolic syndrome (5 missing waist circumference, 37 triglyceride levels, 39 HDL cholesterol levels, 31 glucose levels), only 684 participants were available for analysis.

Data are described as mean and standard deviation for normally, or as median and corresponding 25^th ^and 75^th ^centiles for clearly non-normally distributed variables. Counts and proportions are reported for categorical variables. We studied cardiac structural and functional abnormalities among participants according to the number of components of the metabolic syndrome. The association between the degree of the metabolic syndrome and cardiac structural and functional abnormalities was assessed by calculating mean values of the dependent cardiac variables by linear regression, adjusting first for age and gender, then for 10-year predicted risk of coronary heart disease by Framingham risk score (continuous variable) and finally for age, gender and systolic blood pressure, considering that the reported association might be explained by increasingly high blood pressure. Likewise, adjusted proportions of categorised cardiac variables were estimated using logistic regression by applying population averages of the cofactors to the regression coefficients derived in the regression model. To analyze a graded effect, we included the concurrently present number of syndrome components as a continuous independent variable in the multiple regression models.

## Results

Table 1 contains the characteristics of the study sample. The metabolic syndrome was present in 19.7% of all participants and it was clearly more common among women than men. High blood pressure was by far the most prevalent single component of the metabolic syndrome and affected about 75% of all participants. Among subjects with high blood pressure according to the ATPIII definition of the metabolic syndrome, 80% (415 out of 519) had hypertension defined by blood pressure higher than or equal to 140/90 mmHg or using anti-hypertensive drugs. Men had a higher predicted risk of coronary heart disease, according to Framingham prediction score. Cardiac abnormalities were also fairly common in this sample and heart failure stage C affected 8.4% of females and 5.2% of males.

Concurrence of various components of the metabolic syndrome increased significantly with age and was more frequently found in women. It was also significantly and strongly associated with predicted 10-year risk of coronary heart disease by the Framingham score.

There was a positive association between the degree of the metabolic syndrome – assessed as number of concurrently present components – and parameters of cardiac structure and function, with a consistent and statistically significant trend for all cardiac variables considered (Table 2). These analyses were adjusted for age and gender. Parameters of left ventricular geometry patterns, left atrial diameter and diastolic dysfunction maintained this trend when taking into account the 10-year predicted risk of coronary heart disease by the Framingham score as an independent variable, while LVSD did not (Table 3). Of note, the association between stage C of heart failure and degree of metabolic syndrome showed borderline significance. Since the Framingham risk score can be computed only for subjects aged less than 75 years and with no coronary heart disease, the data on Table 3 refer only to 541 subjects who fulfil these criteria. In order to assess whether the difference between adjusting for age and gender (Table 2) and adjusting for Framingham risk score (Table 3, in bold font) was explained by adjustment to different covariates or by the subsample that was being used, we also present data on this subsample (aged < 75 years, with no coronary heart disease) adjusting only for age and gender (Table 3, square brackets). The difference in the association between LVSD and number of features of the metabolic syndrome was in fact due to adjustment for Framingham risk score. The adjusted prevalence of LVSD increased with increasing degree of metabolic syndrome, but the association was not statistically significant when adjusting for Framingham risk score.

**Figure 1:**
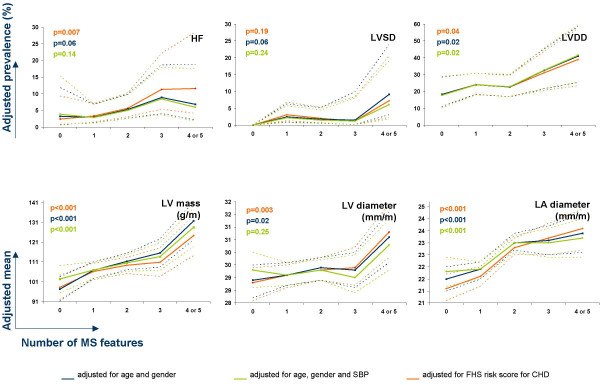
Adjusted prevalence of stage C of heart failure (HF), left ventricular systolic dysfunction (LVSD) and left ventricular diastolic dysfunction (LVDD) according to number of features of the metabolic syndrome, estimated by multiple linear regression (upper panel). Adjusted mean of left ventricular (LV) mass, LV diameter and left atrial (LA) diameter, all indexed to height, according to number of features of the metabolic syndrome estimated by multiple logistic regression (lower panel). In each chart, three models are presented: adjusting for age and gender (blue line), adjusting for the predicted 10-year risk of coronary heart disease by the Framingham risk score (orange) and adjusting for age, gender and systolic blood pressure (green). The dotted lines represent 95% confidence intervals of estimates. P values are for linear trend and were estimated by running the same models with the number of features of the metabolic syndrome as a continuous variable.

The prevalence of left ventricular diastolic dysfunction, and the mean left ventricular mass, left ventricular diameter and left atrial diameter increased significantly with the number of features of the metabolic syndrome when additionally adjusting for systolic blood pressure as a continuous variable (Figure 1).

## Discussion

In this study, we found an association between systolic and diastolic dysfunction and the degree of metabolic syndrome, with the frequency and/or the severity of systolic and diastolic dysfunction increasing with the number of features of the metabolic syndrome. Importantly, early asymptomatic stages of cardiac dysfunction increased progressively with the severity of the metabolic syndrome, independently of systolic blood pressure.

Previous studies demonstrated an association between insulin resistance and heart failure [29]. In a nested case-control study in Swedish elderly men [7], factors associated with insulin resistance (heart rate, serum proinsulin, a high proportion of dihomogammalinolenic acid in serum cholesterol esters and hypophosphataemia) were associated with left ventricular systolic dysfunction after 20-year follow-up, independently of ischaemic heart disease, hypertension and medications. In the Strong Heart Study [30], American Indians with the metabolic syndrome had greater left ventricular dimension, mass, relative wall thickness and left atrial diameter, a higher prevalence of left ventricular hypertrophy, and lower ejection fraction and mitral E/A ratio. In a cross-sectional analysis within the ARIC Study [31], the degree of metabolic syndrome clustering was strongly related to LV mass and wall thickness in black women and men. This association was not observed for chamber size, suggesting that there was a specific effect on myocardial thickening but not dilation. Our study describes a population-based sample of Caucasian men and women, in a country with low prevalence of ischaemic heart disease and high prevalence of hypertension and stroke. Recently we showed that the prevalence of heart failure is not lower than in other western countries [16]. We thoroughly characterised participants in terms of cardiac structure and function. The statistical association with increasing number of features of metabolic syndrome can be explained by the increasing impact of multiple independent risk factors and does not necessarily mean that there is synergism. Given the tendency of individual factors to aggregate, the prevalence of each component in isolation was very low, except for high blood pressure as shown in Table 1. Therefore, it was not possible to estimate the sole effect of each factor, in comparison with the absence of all factors. This would be necessary to truly assess an interaction between individual factors. Given that increasing concurrence of factors of the metabolic syndrome might only be a proxy for higher blood pressure, it is a strength of this study that the reported associations were not explained by blood pressure level.

From the clinical and public health perspective, it has been questioned whether metabolic syndrome improves cardiovascular risk prediction, beyond previously used tools such as the Diabetes Predicting Model for type 2 diabetes or Framingham risk score for coronary heart disease [12-14], [32-34]. Some studies have assessed whether the metabolic syndrome predicts the risk of cardiovascular diseases or a surrogate such as subclinical atherosclerosis [35,36]. In the majority of these studies, however, the outcome with which the metabolic syndrome was to be related was atherosclerotic vascular disease, either coronary heart disease alone or stroke. When assessing cardiac structure and function, one must keep in mind that coronary heart disease is not the only determinant of systolic and diastolic dysfunction. When adjusting for Framigham risk score, we are in fact assessing the effect of features of the metabolic syndrome not considered in the score (obesity and triglycerides) as well as abnormalities of carbohydrate metabolism that are not severe enough to establish the diagnosis of diabetes (impaired fasting glucose). Moreover, if there is increasing insulin resistance with increasing degree of metabolic syndrome, there may additionally be a mitogenic stimulus for cardiac hypertrophy. It is not very surprising, therefore, that particularly LV mass, wall thickness and, probably in consequence, atrial diameter were the cardiac parameters that remained significantly associated with increasing severity of metabolic syndrome even when adjusting for the Framingham risk score.

In comparison with previous studies, this study additionally shows that the association is not fully explained by the level of blood pressure, and that the metabolic syndrome may help predict an increased cardiovascular risk beyond that predicted by the more frequently used tool Framingham risk score.

The main limitation of the present study is the relatively small sample size expectedly leading to few outcomes in certain categories, such as left ventricular systolic dysfunction, and weakening assessment of interactions between the factors of the metabolic syndrome, which would be most relevant in trying to document synergistic effects. The cross-sectional design is also not the ideal approach to assess causality. Advanced cachectic heart failure patients have a particularly high fatality rate and are most likely underrepresented in a dwellers sample like ours (length bias) and less likely to participate if selected (due to functional impairment). An overestimation of the strength of the association with heart failure could also result from the "reverse epidemiology" of cardiovascular risk factors [37], because once overt heart failure is installed obesity, hypertension and hypercholesterolaemia are associated with increased survival. The assessment of early stages of the outcome up to overt heart failure instead of the final outcome should avoid this bias and is a strength of our study. Restriction to participants who do not take any antihypertensive drug, statin, insulin or antidiabetic would result in a probably differential reduction of the sample size to 413 participants, either because those that are medicated have more "severe" metabolic syndrome or on the contrary because they are more health concerned and under closer surveillance. The assessment of the association between the metabolic syndrome and cardiac structure and function in this group would be severely biased (selection bias). Thus, we can only speculate that if medications attenuate the cardiovascular effects of the metabolic syndrome, our estimate of effect is likely conservative.

## Conclusion

In summary, symptomatic heart failure and several cardiac structural and functional abnormalities regardless of symptoms increased progressively with increasing degree of metabolic syndrome. This association was independent of 10-year predicted risk of coronary heart disease by Framingham risk score for indirect indices of diastolic dysfunction but not systolic dysfunction.

## Competing interests

The author(s) declare that they have no competing interests.

## Authors' contributions

AA, PB, ACS, CAL, HB have made substantial contributions to conception and design

AA, PB, PBA, ACS have made substantial contributions to acquisition of data

AA, PB, CAL, HWH, HB have made substantial contributions to analysis and interpretation of data

AA, HWH, HB have been involved in drafting the manuscript and revising it critically for important intellectual content

PBA, ACS, CAL have been involved in revising the manuscript critically for important intellectual content

All authors have given final approval of the version to be published.

## Pre-publication history

The pre-publication history for this paper can be accessed here:



## Figures and Tables

**Table 1:** Characteristics of the study sample and prevalence of the metabolic syndrome and its defining features.

	**Women n = 416**	**Men n = 268**
Age (years), mean (standard deviation)	61 (10)	62 (11)

Metabolic syndrome, n(%)	97 (23.3)	38 (14.2)

Number of features of the metabolic syndrome, n(%)		
0	59 (14.2)	35 (13.1)
1	123 (29.6)	106 (39.6)
High blood pressure	92 (60.9)	11 (23.9)
High triglycerides	4 (6.3)	11 (23.9)
Low HDL-cholesterol	20 (25.3)	5(12.5)
Waist circumference	5 (7.8)	0
High fasting serum glucose	2 (3.3)	3(7.9)
2	137 (32.9)	89 (33.2)
3	55 (13.2)	24 (9.0)
4–5	42 (10.1)	14 (5.2)

Features of the metabolic syndrome, n(%)		
High blood pressure (≥ 130/85 mmHg)	307 (73.8)	212 (79.1)
High triglycerides (≥ 150 mg/dl)	90 (21.6)	79 (29.5)
Low HDL-cholesterol (< 50 mg/dl women; < 40 mg/dl men)	66 (15.9)	23 (8.6)
Waist circumference > 88 cm women or > 102 cm men	215 (51.7)	45 (16.8)
High fasting serum glucose (≥ 110 mg/dl)	60 (14.4)	55 (20.5)

Coronary heart disease, n(%)	34 (8.2)	30 (11.2)

10-year risk of CHD (FHS risk score)*		
< 5%	104 (30.7)	20 (9.7)
5–10%	145 (43.3)	58 (28.2)
10–15%	57 (17.0)	57 (27.7)
≥ 15%	30 (9.0)	71 (34.5)

**LVSD, n(%)**	10 (2.4)	20 (7.5)
LV diameter/height (mm/m), median (interquartile range)	30.1 (28.3–32.2)	29.1 (27.1–31.2)
LV mass/height (g/m), median (interquartile range)	103.7 (86.4–124.5)	112.5 (92.0–136.1)
LV mass/height^2.7 ^(g/m^2.7^), median (interquartile range)	50.2 (40.8–61.0)	46.6 (37.7–57.2)
LV mass/FFM (g/kg), median (interquartile range)	3.97 (3.28–4.71)	3.51 (2.87–4.20)
Posterior wall/height (mm/m), median (interquartile range)	5.5 (5.1–6.2)	5.4 (4.8–6.0)
Interventricular septum/height (mm/m), median (interquartile range)	6.2 (5.6–6.8)	5.9 (5.4–6.6)
Relative wall thickness, median (interquartile range)	0.37 (0.33–0.42)	0.37 (0.33–0.41)
Left atrium/height (mm/m), median (interquartile range)	23.0 (21.3–24.8)	21.9 (20.5–23.7)
E wave/A wave (peak velocity), median (interquartile range)	0.95 (0.78–1.20)	0.94 (0.77–1.16)
Diastolic dysfunction, n(%)	97 (24.1)	74 (29.0)

Stage C of heart failure (symptomatic cardiac dysfunction), n(%)	35 (8.4)	14 (5.2)

*Valid values available for 541 participants.

CHD, coronary heart disease; FHS, Framingham Heart Study; LVSD, left ventricular systolic dysfunction; LV, left ventricle; FFM, fat-free mass

**Table 2:** Cardiac structure and function parameters according to number of features of metabolic syndrome, adjusted for age and gender.

	Number of features of the metabolic syndrome					
		
	0	1	2	3	4–5	p (trend)
	n = 94	n = 229	n = 226	n = 79	n = 56	
**Adjusted prevalence (95%CI)**						
Stage C of heart failure	2.4 (0.6–9.2)	4.0 (2.2–7.3)	4.7 (2.7–8.3)	8.1 (4.0–15.7)	10.5 (5.1–20.5)	0.007
LVSD	0.0 (0.0-0.0)	2.7 (1.3–5.6)	3.0 (1.5–6.0)	2.7 (0.8–8.5)	7.1 (2.9–16.6)	0.02
Diastolic dysfunction	20.8 (13.4–30.6)	23.9 (18.7–30.1)	25.7 (20.3–32.0)	30.2 (20.9–41.6)	36.0 (24.1–49.9)	0.03
Coronary heart disease	3.6 (1.2–10.6)	6.5 (4.0–10.5)	8.1 (5.2–12.4)	10.0 (5.2–18.6)	18.4 (10.3–30.7)	0.002

**Adjusted mean (95%CI)**						
LV diameter/height (mm/m)	29.4 (28.8–30.0)	29.7 (29.3–30.1)	29.9 (29.5–30.3)	29.9 (29.2–30.6)	30.9 (30.1–31.6)	0.008
LV mass/height (g/m)	100.1 (93.5–106.8)	109.0 (104.8–113.3)	113.8 (109.7–118.0)	120.0 (112.8–127.2)	129.9 (121.3–138.5)	< 0.001
LV mass/height^2.7 ^(g/m^2.7^)	45.9 (42.8–49.1)	49.7 (47.7–51.7)	52.1 (50.1–54.1)	54.5 (51.1–58.0)	59.4 (55.3–63.4)	< 0.001
LV mass/FFM (g/kg)	3.66 (3.44–3.89)	3.92 (3.78–4.07)	3.97 (3.82–4.11)	4.08 (3.83–4.33)	4.39 (4.09–4.68)	< 0.001
Posterior wall/height (mm/m)	5.3 (5.2–5.5)	5.5 (5.4–5.6)	5.6 (5.5–5.7)	5.8 (5.6–6.0)	5.8 (5.6–6.1)	< 0.001
Interventricular septum/height (mm/m)	5.8 (5.6–6.0)	6.1 (5.9–6.2)	6.2 (6.1–6.4)	6.4 (6.2–6.6)	6.6 (6.4–6.9)	< 0.001
Relative wall thickness	0.37 (0.35–0.38)	0.38 (0.37–0.38)	0.38 (0.37–0.39)	0.39 (0.37–0.40)	0.38 (0.36–0.40)	0.05
Left atrium/height (mm/m)	22.0 (21.4–22.6)	22.3 (22.0–22.7)	23.5 (23.1–23.8)	23.5 (22.9–24.1)	24.1 (23.4–24.8)	< 0.001
E wave/A wave (peak velocity)	1.06 (1.00–1.12)	1.04 (1.00–1.08)	0.97 (0.93–1.01)	0.91 (0.85–0.98)	0.94 (0.86–1.02)	< 0.001

CI, confidence interval; LVSD, left ventricular systolic dysfunction; LV, left ventricle; FFM, fat-free mass.

**Table 3:** Cardiac structure and function parameters according to degree of metabolic syndrome, adjusted for 10-year risk of CHD predicted by Framingham Heart Study risk score, in 541 participants aged < 75 years and with no CHD (bold font).

	Number of features of the metabolic syndrome					
		
	0	1	2	3	4–5	
	n = 86	n = 185	n = 176	n = 61	n = 33	p (trend)
**Adjusted prevalence (95%CI)**						
Stage C of heart failure	**2.4 (0.6–9.3)** [3.2 (0.8–11.9)]	**3.3 (1.5–7.1)** [3.0 (1.3–6.8)]	**5.7 (3.1–10.2)** [5.3 (2.7–9.9)]	**11.3 (5.4–22.1)** [8.9 (4.0–18.7)]	**11.6 (4.1–28.6)** [6.8 (2.2–18.9)]	**0.007** 0.06
LVSD	**0.0 (0.0–0.0)** [0.0 (0.0–0.0)]	**3.1 (1.3–6.8)** [2.5 (1.0–6.3)]	**2.0 (0.7–5.3)** [1.8 (0.6–5.2)]	**1.2 (0.2–8.4)** [1.5 (0.2–9.9)]	**7.3 (2.3–20.5)** [9.1 (3.1–23.9)]	**0.19** 0.06
Diastolic dysfunction	**18.3 (11.2–28.5)** [18.3 (11.2–28.5)]	**24.1 (18.4–30.9)** [23.9 (18.1–30.9)]	**22.7 (16.9–29.6)** [22.8 (17.0–30.0)]	**31.1 (20.7–43.9)** [32.4 (21.7–45.3)]	**39.1 (23.5–57.3)** [41.0 (25.4–58.6)]	**0.04** 0.02

**Adjusted mean (95%CI)**						
LV diameter/height (mm/m)	**29.1 (28.4–29.7)** [29.2 (28.5–29.8)]	**29.4 (28.9–29.8)** [29.4 (29.0–29.9)]	**29.6 (29.2–30.1)** [29.7 (29.2–30.1)]	**29.7 (29.0–30.5)** [29.6 (28.9–30.3)]	**31.1 (30.1–32.2)** [30.9 (29.9–31.9)]	**0.003** 0.02
LV mass/height (g/m)	**98.3 (92.1–104.5)** [97.4 (91.3–103.6)]	**106.3 (102.1–110.4)** [106.8 (102.6–111.0)]	**109.4 (105.2–113.5)** [111.4 (107.2–115.5)]	**110.9 (103.8–118.1)** [115.6 (108.5–122.7)]	**124.3 (114.3–134.3)** [131.8 (122.2–141.4)]	**< 0.001** < 0.001
LV mass/height^2.7 ^(g/m^2.7^)	**43.5 (40.6–46.5)** [44.6 (41.7–47.5)]	**47.7 (45.7–49.6)** [48.7 (46.7–50.7)]	**49.8 (47.8–51.7)** [50.9 (49.0–52.9)]	**51.4 (48.0–54.8)** [52.1 (48.8–55.4)]	**58.8 (54.0–63.5)** [59.5 (54.9–64.0)]	**< 0.001** < 0.001
LV mass/FFM (g/kg)	**3.47 (3.25–3.69)** [3.58 (3.37–3.79)]	**3.75 (3.60–3.89)** [3.85 (3.70–3.99)]	**3.79 (3.64–3.93)** [3.88 (3.74–4.02)]	**3.92 (3.67–4.17)** [3.92 (3.68–4.16)]	**4.41 (4.06–4.76)** [4.37 (4.04–4.70)]	**< 0.001** < 0.001
Posterior wall/height (mm/m)	**5.2 (5.0–5.4)** [5.3 (5.1–5.5)]	**5.4 (5.3–5.6)** [5.5 (5.4–5.6)]	**5.5 (5.4–5.6)** [5.6 (5.5–5.7)]	**5.7 (5.4–5.9)** [5.7 (5.5–5.9)]	**5.9 (5.6–6.2)** [5.9 (5.7–6.2)]	**< 0.001** < 0.001
Interventricular septum/height (mm/m)	**5.6 (5.4–5.8)** [5.7 (5.5–5.9)]	**6.0 (5.8–6.1)** [6.0 (5.9–6.2)]	**6.1 (6.0–6.3)** [6.2 (6.1–6.4)]	**6.3 (6.0–6.5)** [6.3 (6.1–6.6)]	**6.4 (6.1–6.8)** [6.5 (6.2–6.8)]	**< 0.001** < 0.001
Relative wall thickness	**0.36 (0.35–0.38)** [0.37 (0.35–0.38)]	**0.37 (0.36–0.38)** [0.38 (0.37–0.39)]	**0.37 (0.36–0.38)** [0.38 (0.37–0.39)]	**0.38 (0.36–0.40)** [0.39 (0.37–0.40)]	**0.39 (0.36–0.41)** [0.39 (0.37–0.41)]	**0.07** 0.07
Left atrium/height (mm/m)	**21.4 (20.9–22.0)** [21.8 (21.3–22.3)]	**21.9 (21.5–22.3)** [22.2 (21.8–22.5)]	**23.1 (22.8–23.5)** [23.3 (23.0–23.7)]	**23.5 (22.8–24.1)** [23.4 (22.8–24.0)]	**23.9 (23.0–24.7)** [23.7 (22.9–24.5)]	**< 0.001** < 0.001
E wave/A wave (peak velocity)	**1.09 (1.02–1.16)** [1.04 (0.98–1.10)]	**1.05 (1.00–1.09)** [1.01 (0.97–1.05)]	**1.01 (0.97–1.06)** [0.96 (0.92–1.01)]	**0.94 (0.87–1.02)** [0.91 (0.84–0.98)]	**1.00 (0.89–1.11)** [0.96 (0.86–1.06)]	**0.01** 0.004

Data in square brackets are adjusted for age and gender, in the same subsample of participants aged < 75 years and with no CHD (n = 541). CHD, coronary heart disease; CI, confidence interval; LVSD, left ventricular systolic dysfunction; LV, left ventricle; FFM, fat-free mass.
